# Evaluation of Performance in Colon Capsule Endoscopy Reading by Endoscopy Nurses

**DOI:** 10.1155/2021/8826100

**Published:** 2021-04-28

**Authors:** Yukiko Handa, Konosuke Nakaji, Kayo Hyogo, Makiko Kawakami, Tomomi Yamamoto, Akiko Fujiwara, Rika Kanda, Motoyasu Osawa, Osamu Handa, Hiroshi Matsumoto, Eiji Umegaki, Akiko Shiotani

**Affiliations:** ^1^Division of Gastroenterology, Department of Internal Medicine, Kawasaki Medical School, Kurashiki City, Okayama, Japan; ^2^Department of Internal Medicine, Aishinkai Nakae Hospital, Wakayama, Japan; ^3^Nursing Department, Aishinkai Nakae Hospital, Wakayama, Japan; ^4^Nursing Department, Kawasaki Medical School Hospital, Kurashiki City, Okayama, Japan

## Abstract

**Background:**

Although there are papers reporting on the accuracy of colon capsule endoscopy (CCE) compared with colonoscopy (CS), there are few reports on the detection rates of significant lesions by endoscopy nurses. We previously reported no significant difference in the detection rates for small bowel capsule endoscopy (SBCE) images among two well-trained physicians and one expert nurse.

**Objective:**

To evaluate the reading time and detection rate of the significant lesions of CCE images among novice and trained expert endoscopy nurses and novice physicians.

**Methods:**

CCE videos of 20 consecutive patients who performed both CCE and CS with clinically significant localized lesions were selected. Two trained expert endoscopy nurses, untrained two novice physicians, and novice three endoscopy nurses reviewed CCE videos. The detection rate of the lesions and reading time were compared among the three groups and were evaluated by comparison between the first and the second 10 videos.

**Results:**

The median reading time was the shortest (19 min) in the trained expert endoscopy nurses and the longest (45 min) in the novice nurses. The number of thumbnails tended to be more in the trained expert endoscopy nurses in the first 10-video reading. Although the detection rates of small polyps (<5 mm) were significantly lower (46.5%, *p*=0.025) in the novice nurses compared to the others, they were improved (35.2% to 63.5%, *p*=0.015) in the second 10 videos. The detection rates of tumor lesions by either one of two trained expert endoscopy nurses were higher compared to those by each novice physician.

**Conclusions:**

The trained expert endoscopy nurses for CCE reading can reduce physician's time and improve the diagnostic yield.

## 1. Introduction

Colorectal cancer (CRC) screening is recommended to individuals over 50 years of age according to the guideline [1], and demands of CRC screening have increased. There are several modalities of colorectal cancer (CRC) screening, such as fecal occult blood testing, colonoscopy (CS), and CT colonography [2]. Screening for CRC has become more widespread reducing the CRC mortality rate in the USA; however, the CRC mortality rate is still increasing and the participation rates in CRC screening are low in Japan. Colon capsule endoscopy (CCE) is a new, minimally invasive, painless endoscopic technique which visualizes the entire colon without sedation and air insufflation [3]. The availability of CCE has been reported to increase adherence to CRC screening. Although a recent paper indicated the limitation of the diagnostic capability of CCE for advanced CRC by the incompletion of the CCE procedure, the sensitivities of colon polyps larger than ≥5 mm using second-generation CCE (CCE-2) technology have been reported to be 84–94% [4–7].

However, CCE reading is a time-consuming task and interpretation of images is highly subjective. Moreover, no formalized training system for CCE and no standardized system to assess CCE reading competence have been established. Training on CCE video reading is generally performed as a lecture on basic information and a hands-on seminar using several clinical case videos. We previously reported no significant difference in the detection rates for small bowel capsule endoscopy (SBCE) images among two well-trained physicians and one expert nurse. A trained nonphysician assistant can reduce physician's time and improve the diagnostic yield of SBCE [8, 9]. Moreover, a recent meta-analysis showed that properly trained physician extenders and/or specialist nurses could replace physicians in the SBCE reading [10].

Therefore, training assistants in interpreting CCE is thought to save the reading time of physicians. However, there are a few reports on the detection rates of significant lesions in CCE by endoscopy nurses. In the present study, we evaluated the reading time and detection rate of 20 CCE videos containing significant tumor lesions assessed by novice nurses, novice physicians, and trained expert endoscopy nurses.

## 2. Methods

This was a study evaluating previously obtained CCE-2 videos (Medtronic, Minneapolis, USA) and colonoscopic findings without personal information. CCE and colonoscopy were performed from January 2014 to September 2017 in the Aishinkai Nakae Hospital. The study was approved by both the research Ethical Committee of Kawasaki Medical School, Okayama, Japan (No. 3195) on 14 September 2018 and Aishinkai Nakae Hospital, Wakayama, Japan (No. 012) on 14 July 2018. The study protocol conforms to the ethical guidelines of the 1975 Declaration of Helsinki as reflected in a prior approval by the institution's human research committee.

In this study, two well-trained endoscopy nurses (M. K. and K. H.), three endoscopy nurses (T. Y., A. F., and R. K.) who had no experience in CCE reading, and two physicians (Y. H. and M. O.) reviewed CCE videos separately. The well-trained nurses M. K and K. H. have a board-certified technical assistant of CCE reading and have experience in reviewing about more than 50 CCE and more than 200 small bowel CE procedures. The two physicians have no experience in CCE reading, although both have board-certified endoscopy physician and M. O. has experience in reviewing about more than 100 small bowel CE procedures.

### 2.1. Selection of CCE Procedure

Twenty CCE procedure images of the patients who performed both CCE and CS with clinically significant localized tumor lesions were selected by the well-trained physician (K. N.).

### 2.2. CCE Reading

All investigators separately read videos in a blinded fashion by erasing the patients' names using a dual mode and the frame rate was not fixed (Given Diagnostic Imaging System, Given Imaging), and they marked suspected lesions as thumbnail photographs. The size of polyps was measured by using a graphical interface tool for poly size estimation in RAPID software. For adjudication, at least two reviewers simultaneously evaluated thumbnail photographs by knowledge of the results of CS. After the investigators had reviewed the first 10 videos, they checked the thumbnail corrected images with significant lesions comparing their selected thumbnail images. After learning the correction, the inexperienced readers reviewed the second 10 videos. We compared the detection rate of the lesions and reading time between the first 10 videos and the second in each group to evaluate the improvement of the diagnosis ability after reading 10 videos.

### 2.3. Statistical Analysis

Values were expressed as mean ± SD or median and 25–75% range whichever was appropriate depending on whether the data were normally distributed. Statistical analysis for significant differences except for reading time among the three groups was performed using one-way factorial analysis of variance, and Bonferroni's method provides a pairwise comparison of the means. Statistical analysis for significant differences of reading time among the three groups was performed using the Kruskal–Wallis one-way analysis and expressed as median (5–95% range). The difference between the first half of reading and the second half of reading in each group was evaluated by an unpaired *t*-test. All statistical analyses were performed using SPSS version 25 for Windows (IBM Japan, Ltd., Tokyo, Japan).

## 3. Results

The length of the video clip was from 20 minutes to 4 hours and 7 minutes (the average length: 2 hours and 33 minutes) in the first 10 videos, and in the second 10 videos, from 38 minutes to 5 hours and 19 minutes (the average length: 2 hours and 29 minutes).

### 3.1. Selected Lesions

The selected CCE videos included significant tumor lesions which were 34 lesions of small polyps (less than 5 mm), 35 lesions of large polys (5 mm and larger), one early colon cancer, and one advanced cancer (Table 1). Two small polyps and 3 large polyps were detected by CS, but not detected by CCE, while 6 small lesions and 3 large polyps were missed by only CS.

### 3.2. Reading Time

**Figure 1:**
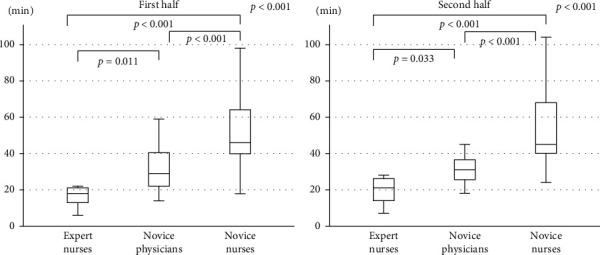
Comparisons of the reading time between the first half of videos and the second half of videos among the three groups (expert nurses, novice physicians, and novice nurses). Horizontal bars are the medians, boxes represent the 25th–75th interquartile ranges, and vertical lines indicate the range of values; *p* values are obtained by the Kruskal–Wallis analysis.

The median reading time was 19 min (95% CI; 6.1–28.0) in the trained expert endoscopy nurses, 30.5 min (95% CI; 18.0–58.5) in the novice physicians, and 45 min (95% CI; 25.1–103.7) in the novice nurses in that order, and the difference was significant (Table 2). A significant difference in the reading time among the three groups was observed in both the first 10 videos and the second (Figure 1). The median reading time by the trained expert endoscopy nurses was the shortest.

### 3.3. Number of Thumbnails

**Figure 2:**
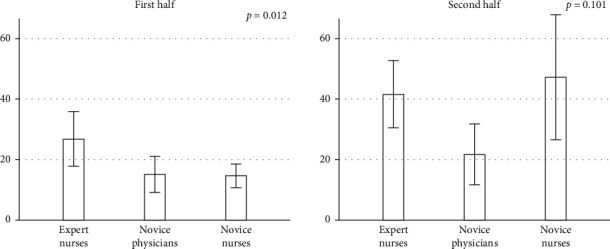
Comparisons of the number of thumbnails between the first half and the second half among the three groups. Data are mean, and error bars represent standard error (SE). *p* values are obtained by one-way factorial analysis of variance.

The mean numbers of thumbnails were tended to be more in the trained expert endoscopy nurses and less in the novice physicians. The mean numbers of thumbnails in the first 10 videos reading were significantly different (*p*=0.012) among the three groups and that of the trained expert endoscopy nurses was most, however, there was no difference in the second half reading (Figure 2). The mean number of thumbnails of the novice nurses was increased (14.6 to 47.2, *p*=0.002) in the second 10 videos.

### 3.4. Detection Rate of Tumor Lesions

**Figure 3:**
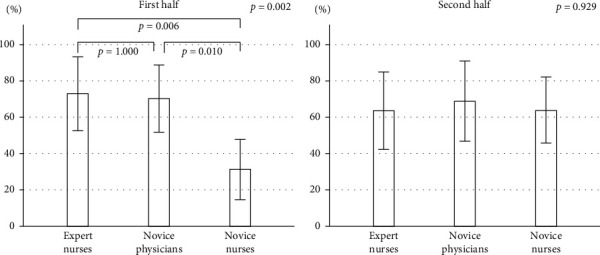
Comparisons of the small polyp detection rate between the first half and the second half among the three groups. Data are mean (%), and error bars represent SE. *p* values are obtained by one-way factorial analysis of variance.

**Figure 4:**
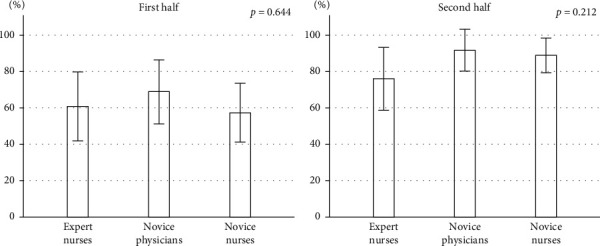
Comparison of the large polyp detection rate between the first half and the second half among three groups. Data are mean (%), and error bars represent SE. *p* values are obtained by one-way factorial analysis of variance.

The detection rates of small polyps less than 5 mm were significantly lower (46.5%, *p*=0.025) in the novice nurses compared to the others, although the detection rates of large polyps were not different. The detection rates of small polyps in the first 10 videos reading were significantly different among the three groups, however, there was no difference in the second 10 videos (Figure 3). The detection rates of small polyps in the novice nurses were improved (35.2% to 63.5%, *p*=0.015) in the second 10 videos. The detection rates of large polyps among the three groups were not different both in the first and second 10 videos, and the detection rate in each group tended to be increased (Figure 4).

### 3.5. Detection Rate of Polyps by Size

**Figure 5:**
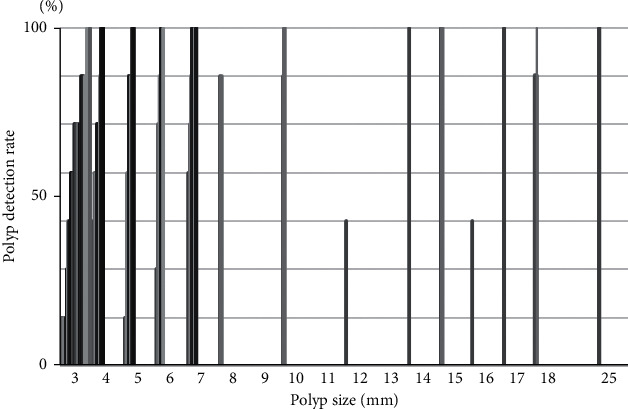
Detection rates of polyps by size.

The detection rates of small polyp were varied, while the detection rates of large polyps were relatively high (Figure 5). However, the detection rates in the two large polyps (Suppl.  1) were 50%, and half the number of readers including one novice physician missed the polyps. The advanced colon cancer was detected by all investigators, but early colon cancer was missed by one reader (Suppl.  2).

### 3.6. Detection Rates by Either One of Two Readers

The detection rates of tumor lesions by either one of two readers were higher compared to those by each novice physician. Either one of two novice physicians detected 97.1% of the large polyps, but only 87.5% small polyps. In contrast, either the trained expert endoscopy nurse or the novice physician detected 100% of the small polyps and 95.5% of the tumor lesions (Table 3).

## 4. Discussion

In the comparison among the trained expert endoscopy nurses, the novice nurses, and the novice physicians, the detection rates of small polyps less than 5 mm were significantly lower (46.5%, *p*=0.025) in the novice nurses compared to the others, although the detection rates of large polyps were not different. The detection rates of small polyps in the novice nurses were improved (35.2% to 63.5%, *p*=0.015), in the second 10 videos after the investigators had reviewed first 10 videos. However, the detection rate of larger polyps was higher even in the first half of the study. Therefore, the polyp size seems to be the important factor affecting detection rate.

Hosoe et al. [11] previously evaluated the learning curve for reading a total of 45 CE videos and indicated that experience of approximately 20 CE readings can be considered as the first step to becoming an expert. Watabe et al. [12] conducted a study evaluating the electronic learning system for CCE (ELCCE), which was originally designed for the members of the Japanese Association for Capsule Endoscopy (JACE). ELCCE including nearly 30 actual clinical CCE videos was confirmed to be useful and effective for improving CCE reading competence. Experience of more than 20 CCE reviews can be considered as the first step to become the level that they can support in the interpretation of CCE. The study recruited only endoscopists, however, our data supported that ELCCE seems to be useful and effective for nurses. Two previous studies investigating the diagnostic yield of SBCE reported that an endoscopy nurse picked up 93% to 94% of the clinically significant lesions of SBCE detected by physicians [13, 14]. In our previous SBCE studies, the detection rates of small bowel injuries in patients taking aspirin in the trained nurse were similar to those in the trained physicians and were superior to those in the physician with limited capsule experience [8, 9].

There were previous studies indicating moderate interobserver discrepancies in the interpretation of CE [15, 16]. Therefore, the diagnostic miss rate in reading images of CE is likely to increase, if only a single investigator reads. Lai et al. reported that there are interobserver variations in the interpretation of CCE results among experienced reviewers. They indicated that a second reading by an experienced viewer improves the diagnostic accuracy of the procedure. In the present CCE study, the number of polyps detected by either of the two readers was higher compared to one reader. Pre-evaluation by at least 2 trained readers not limited to physicians could reduce diagnostic miss rates and time-consuming by physicians.

The mean number of thumbnails tended to be more in the trained expert endoscopy nurses and less in the novice physicians. The mean numbers of thumbnails of the second 10 videos were increased in the novice nurses after learning the correction and were not different among the three groups. The median reading time was the shortest (19 min) in the trained expert endoscopy nurses and the longest (45 min) in the novice nurses, while the median reading times between the first half and the second half of videos were not different in each group. Further training could reduce the reading time in novice readers.

Substantial progress has recently been made in artificial intelligence (AI) in gastroenterology, mainly focusing on AI assisted-endoscopy [17–19]. A recent review demonstrated that many studies have assessed the use of AI to detect malignant lesions and to facilitate the analysis of inflammatory and other nonmalignant lesions and small bowel bleeding based on images collected during SBCE. Most studies indicated excellent accuracy which was higher than 90% [20]. In contrast, there are a few studies investigating AI to improve diagnostic accuracy in case of colorectal polyps based on CCE images [21–23]. High-quality dataset for AI development is still lacking and most evidence to develop machine learning (ML) algorithms comes from preclinical studies without applications used in clinical practice.

Several limitations should be considered when interpreting our results. First, this was a retrospective study, and the numbers of investigators and examined videos were small. The number of lesions detected only by CCEs was too small to clarify the clinical significance of those lesions. The frame rate was not fixed among investigators. Moreover, we did not assess the cleansing levels of the colon by bowel preparation that may have influenced our diagnostic yield.

In conclusion, it is possible that the well-trained endoscopy nurses can detect the significant lesions as well as the trained physicians. The trained expert endoscopy nurses for CCE reading can reduce physician's time and improve the diagnostic yield. Developing further improved software and the introduction of AI for reading CCE are required.

## Figures and Tables

**Table 1:** Significant tumor lesions detected colonoscopy (CS) and/or CCE.

Findings of polyps or tumors	First half of CCE	Second half of CCE	Total
*Lesions*			
Polyps < 5 mm	20	14	34
≧5 mm	20	15	35
Early colon cancer (18 mm)	0	1	1
Advanced colon cancer (25 mm)	0	1	1

*Pointed out only in CS*			
<5 mm	2	0	2
≧5 mm	2	1	3

*Pointed out only in CCE*			
<5 mm	3	3	6
≧5 mm	2	1	3

**Table 2:** Performance in colon capsule endoscopy reading among the three groups.

	Expert nurses	Novice physicians	Novice nurses	*p*
Reading time (min) median (95% CI)	19.0 (6.1 – 28.0)	30.5 (18.0 – 58.5)	45.0 (25.1 – 103.7)	<0.001^a^
Number of thumbnails (SD)	34.2 (23.6)	18.4 (18.6)	30.9 (43.5)	0.072^b^
Small polyp detection rate % (SD)	68.5 (42.5)	69.5 (41.1)	46.5 (46.6)	0.025^b^
Large polyp detection rate % (SD)	68.2 (37.1)	80.2 (31.2)	73.1 (35.9)	0.382^b^

^a^ *p* values were calculated by Kruskal–Wallis one-way analysis. ^b^One-way analysis of variance.

**Table 3:** Detection rates of polyps or/and tumors by the novice physicians or expert nurses.

	Small polyp *n* = 32	Large polyp *n* = 34	Total *n* = 66
Novice physician A	27 (84.4%)	30 (88.2%)	57 (86.4%)
Novice physician B	21 (65.6%)	31 (91.2%)	52 (78.8%)
Two novice physicians	28 (87.5%)	33 (97.1%)	61 (92.4%)
Two expert nurses^*∗*^	31 (96.9%)	31 (91.2%)	62 (93.9%)
One expert nurse and one novice physician^*∗*^	32 (100%)	31 (91.2%)	63 (95.5%)

^*∗*^The detection rates of tumor lesions by either one of the two readers.

## Data Availability

The data used to support the findings of this study are available from the corresponding author upon request.

## Supplementary Materials

Supplementary Materials Suppl. 1: polyps with low detection rates reviewed by CCE. Polyp size was 12 mm (a) and 16 mm (b). Suppl. 2: an advanced colon cancer (a) and an early colon cancer (b) reviewed by CCE.

## References

[1] Lieberman D. A., Rex D. K., Winawer S. J., Giardiello F. M., Johnson D. A., Levin T. R. (2012). Guidelines for colonoscopy surveillance after screening and polypectomy: a consensus update by the US Multi-Society Task Force on Colorectal Cancer. *Gastroenterology*.

[2] Stoop E. M., de Haan M. C., de Wijkerslooth T. R. (2012). Participation and yield of colonoscopy versus non-cathartic CT colonography in population-based screening for colorectal cancer: a randomised controlled trial. *The Lancet Oncology*.

[3] Eliakim R., Fireman Z., Gralnek I. (2006). Evaluation of the PillCam Colon capsule in the detection of colonic pathology: results of the first multicenter, prospective, comparative study. *Endoscopy*.

[4] Eliakim R., Yassin K., Niv Y. (2009). Prospective multicenter performance evaluation of the second-generation colon capsule compared with colonoscopy. *Endoscopy*.

[5] Spada C., Hassan C., Munoz-Navas M. (2011). Second-generation colon capsule endoscopy compared with colonoscopy. *Gastrointestinal Endoscopy*.

[6] Rex D. K., Lieberman D. A. (2012). A survey of potential adherence to capsule colonoscopy in patients who have accepted or declined conventional colonoscopy. *Journal of Clinical Gastroenterology*.

[7] Saito Y., Saito S., Oka S. (2015). Evaluation of the clinical efficacy of colon capsule endoscopy in the detection of lesions of the colon: prospective, multicenter, open study. *Gastrointestinal Endoscopy*.

[8] Shiotani A., Honda K., Kawakami M. (2011). Evaluation of RAPID 5 Access software for examination of capsule endoscopies and reading of the capsule by an endoscopy nurse. *Journal of Gastroenterology*.

[9] Shiotani A., Honda K., Kawakami M. (2012). Analysis of small-bowel capsule endoscopy reading by using quickview mode. *Journal of Clinical Gastroenterology*.

[10] Yung D. E., Fernandez‐Urien I., Douglas S. (2017). Systematic review and meta‐analysis of the performance of nurses in small bowel capsule endoscopy reading. *United European Gastroenterology Journal*.

[11] Hosoe N., Rey J.-F., Imaeda H. (2012). Evaluations of capsule endoscopy software in reducing the reading time and the rate of false negatives by inexperienced endoscopists. *Clinics and Research in Hepatology and Gastroenterology*.

[12] Watabe H., Nakamura T., Yamada A., Kakugawa Y., Nouda S., Terano A. (2016). Assessment of an electronic learning system for colon capsule endoscopy: a pilot study. *Journal of Gastroenterology*.

[13] Levinthal G. N., Burke C. A., Santisi J. M. (2003). The accuracy of an endoscopy nurse in interpreting capsule endoscopy. *American Journal of Gastroenterology*.

[14] Riphaus A., Richter S., Vonderach M., Wehrmann T. (2009). Capsule endoscopy interpretation by an endoscopy nurse - a comparative trial. *Zeitschrift für Gastroenterologie*.

[15] Lai L. H., Wong G. L. H., Chow D. K. L., Lau J. Y. W., Sung J. J. Y., Leung W. K. (2006). Inter-observer variations on interpretation of capsule endoscopies. *European Journal of Gastroenterology & Hepatology*.

[16] Cave D. R., Fleischer D. E., Leighton J. A. (2008). A multicenter randomized comparison of the Endocapsule and the Pillcam SB. *Gastrointestinal Endoscopy*.

[17] Lahiff C., East J. E. (2017). Endoscopic approach to polyp recognition. *Frontline Gastroenterology*.

[18] Ruffle J. K., Farmer A. D., Aziz Q. (2019). Artificial intelligence-assisted gastroenterology- promises and pitfalls. *American Journal of Gastroenterology*.

[19] Yang Y. J., Bang C. S. (2019). Application of artificial intelligence in gastroenterology. *World Journal of Gastroenterology*.

[20] Le Berre C., Sandborn W. J., Aridhi S. (2020). Application of artificial intelligence to gastroenterology and hepatology. *Gastroenterology*.

[21] Figueiredo P. N., Figueiredo I. N., Prasath S., Tsai R. (2011). Automatic polyp detection in pillcam colon 2 capsule images and videos: preliminary feasibility report. *Diagn Ther Endosc*.

[22] Mamonov A. V., Figueiredo I. N., Figueiredo P. N., Richard Tsai Y.-H. (2014). Automated polyp detection in colon capsule endoscopy. *IEEE Transactions on Medical Imaging*.

[23] Blanes-Vidal V., Baatrup G., Nadimi E. S. (2019). Addressing priority challenges in the detection and assessment of colorectal polyps from capsule endoscopy and colonoscopy in colorectal cancer screening using machine learning. *Acta Oncologica*.

