# Supplementary figures and images for: Evaluation of Performance in Colon Capsule Endoscopy Reading by Endoscopy Nurses
Source: Can J Gastroenterol Hepatol. 2021 Apr 28;2021:8826100. doi: 10.1155/2021/8826100 (PMC8100384; doi:10.1155/2021/8826100)

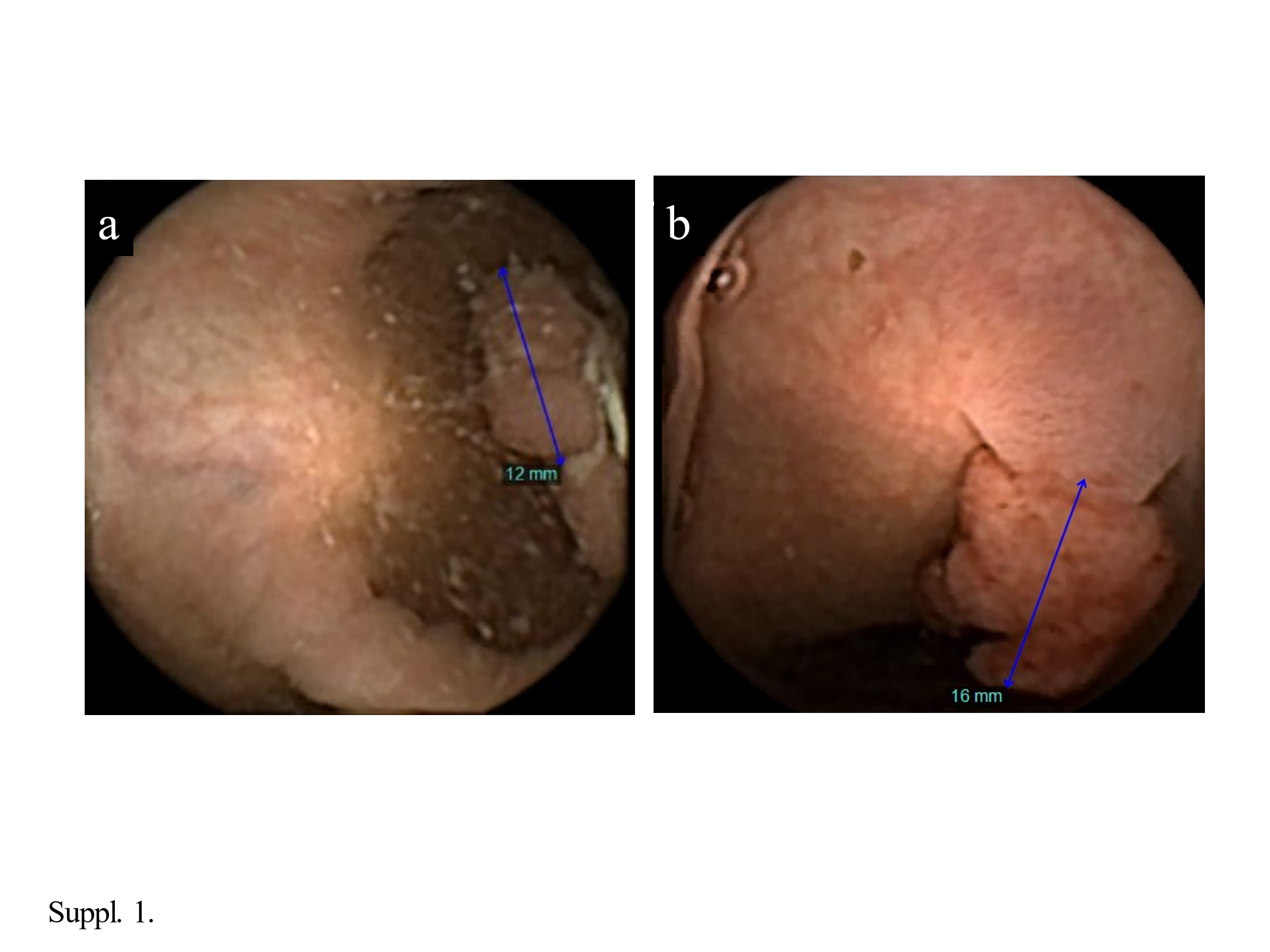


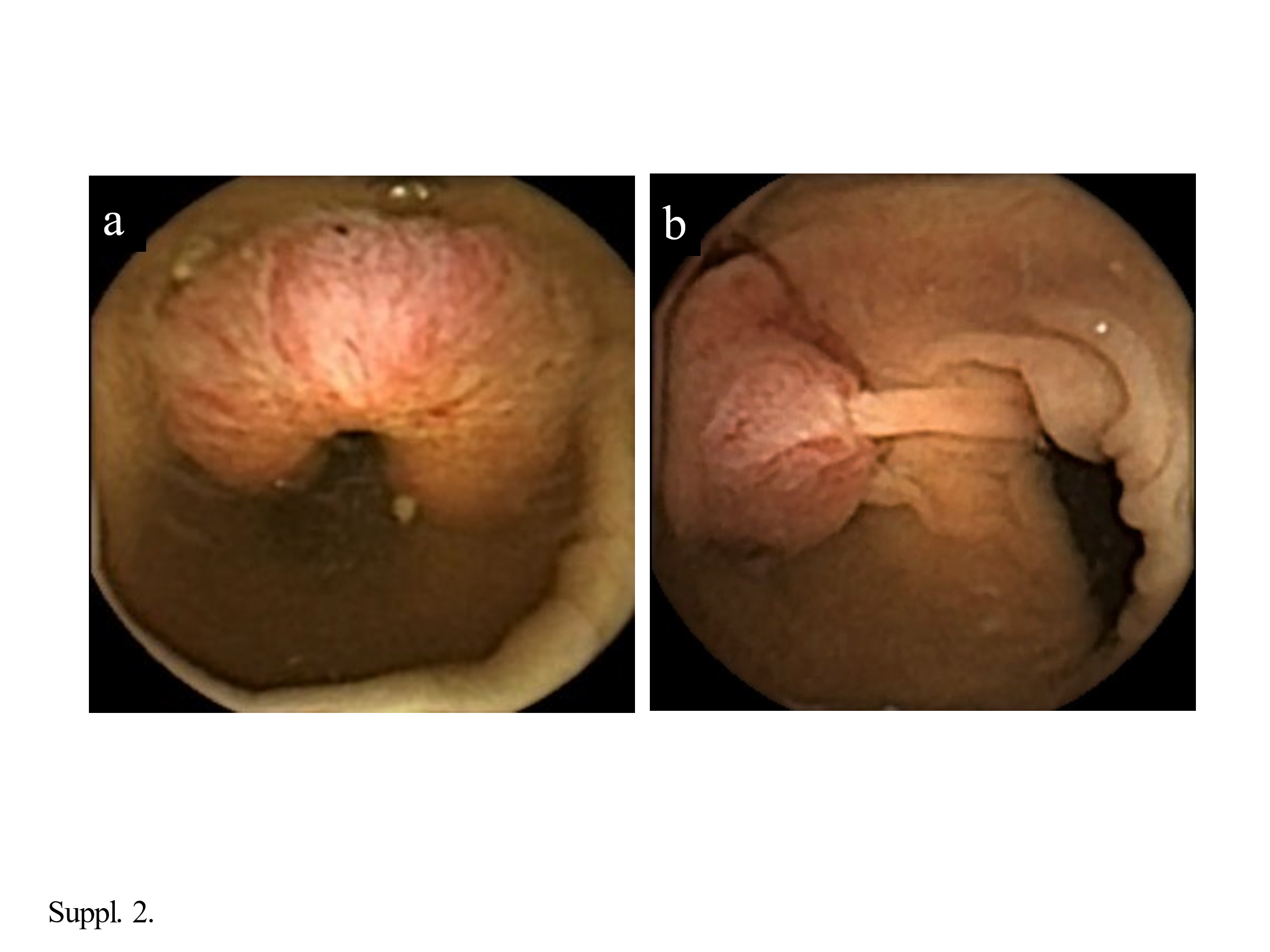

Supplement: Supplementary Materials — Suppl. 1: polyps with low detection rates reviewed by CCE. Polyp size was 12 mm (a) and 16 mm (b). Suppl. 2: an advanced colon cancer (a) and an early colon cancer (b) reviewed by CCE. [file 8826100.f1.docx]
